# Alcohol consumption and risk of dementia: 23 year follow-up of Whitehall II cohort study

**DOI:** 10.1136/bmj.k2927

**Published:** 2018-08-01

**Authors:** Séverine Sabia, Aurore Fayosse, Julien Dumurgier, Aline Dugravot, Tasnime Akbaraly, Annie Britton, Mika Kivimäki, Archana Singh-Manoux

**Affiliations:** 1 Inserm, U1018, Centre for Research in Epidemiology and Population Health, Université Paris-Saclay, France; Hôpital Paul Brousse, Bât 15/16, Villejuif Cedex, France; 2 Department of Epidemiology and Public Health, University College London, UK; 3 Cognitive Neurology Center, Lariboisière-Fernand Widal hospital, AP-HP, Université Paris Diderot, Sorbonne Paris Cité, Paris, France; 4 Inserm U1198, Montpellier, France; University Montpellier, Montpellier, France; EPHE, Paris, France; 5 Department of Psychiatry & Autism Resources Centre, University Research and Hospital Center (CHRU) of Montpellier, Montpellier, France

## Abstract

**Objective:**

To examine the association between alcohol consumption and risk of dementia.

**Design:**

Prospective cohort study.

**Setting:**

Civil service departments in London (Whitehall II study).

**Participants:**

9087 participants aged 35-55 years at study inception (1985/88).

**Main outcome measures:**

Incident dementia, identified through linkage to hospital, mental health services, and mortality registers until 2017. Measures of alcohol consumption were the mean from three assessments between 1985/88 and 1991/93 (midlife), categorised as abstinence, 1-14 units/week, and >14 units/week; 17 year trajectories of alcohol consumption based on five assessments of alcohol consumption between 1985/88 and 2002/04; CAGE questionnaire for alcohol dependence assessed in 1991/93; and hospital admission for alcohol related chronic diseases between 1991 and 2017.

**Results:**

397 cases of dementia were recorded over a mean follow-up of 23 years. Abstinence in midlife was associated with a higher risk of dementia (hazard ratio 1.47, 95% confidence interval 1.15 to 1.89) compared with consumption of 1-14 units/week. Among those drinking >14 units/week, a 7 unit increase in alcohol consumption was associated with a 17% (95% confidence interval 4% to 32%) increase in risk of dementia. CAGE score >2 (hazard ratio 2.19, 1.29 to 3.71) and alcohol related hospital admission (4.28, 2.72 to 6.73) were also associated with an increased risk of dementia. Alcohol consumption trajectories from midlife to early old age showed long term abstinence (1.74, 1.31 to 2.30), decrease in consumption (1.55, 1.08 to 2.22), and long term consumption >14 units/week (1.40, 1.02 to 1.93) to be associated with a higher risk of dementia compared with long term consumption of 1-14 units/week. Analysis using multistate models suggested that the excess risk of dementia associated with abstinence in midlife was partly explained by cardiometabolic disease over the follow-up as the hazard ratio of dementia in abstainers without cardiometabolic disease was 1.33 (0.88 to 2.02) compared with 1.47 (1.15 to 1.89) in the entire population.

**Conclusion:**

The risk of dementia was increased in people who abstained from alcohol in midlife or consumed >14 units/week. In several countries, guidelines define thresholds for harmful alcohol consumption much higher than 14 units/week. The present findings encourage the downward revision of such guidelines to promote cognitive health at older ages.

## Introduction

Excessive alcohol consumption is a leading risk factor for several chronic diseases and mortality.^1^ ^2^ With continuously increasing life expectancy and the expected tripling of dementia prevalence by 2050,^3^ understanding the impact of alcohol consumption on aging outcomes is important.^4^ Moderate alcohol consumption has been suggested to lower the risk of dementia, and the association of alcohol consumption with cognitive outcomes is thought to be J-shaped or U-shaped.^5^ ^6^ ^7^ However, several issues remain unresolved that might explain why alcohol consumption is not listed in the most recent guideline on modifiable risk factors for the prevention of dementia.^8^ Firstly, as most studies rely on face-to-face assessment for dementia diagnosis, people who drop out of the study or die during follow-up are not included in the analyses, resulting in potential bias due to selection in results.^9^ ^10^ This is particularly likely in relation to excessive alcohol consumption, which is known to be associated with greater mortality and drop-out rates.^9^ Secondly, most studies on aging assess alcohol consumption in late life,^5^ ^6^ ^7^ which may not reflect lifetime consumption, and this may be critically important for dementia as it involves neuropathological changes over many years, perhaps decades. The tendency to reduce alcohol consumption at older ages may bias results^11^ and prevent accurate analyses of the quantity of alcohol consumed.^5^ ^7^ Thirdly, most studies use a single assessment of alcohol consumption, which is prone to measurement error. Furthermore, indicators of heavy drinking other than the reported frequency and units of alcohol consumed may add to understanding dementia but are rarely used.^12^ In addition, the mechanisms underlying the association between alcohol and cognitive aging remain unclear.^5^ ^6^ ^7^ Non-moderate alcohol consumption is associated with a higher risk of cardiometabolic disease,^1^ ^13^ ^14^ ^15^ which is itself associated with a higher risk of dementia,^4^ ^16^ suggesting a potential role of these diseases in the association between alcohol consumption and dementia.

To tackle some of these limitations, we used repeat data spanning nearly three decades to investigate the association between alcohol consumption and risk of dementia, assessed through linkage to electronic health records for all participants irrespective of their continued participation in follow-up. We examined associations of dementia with alcohol consumption in midlife, alcohol dependence, hospital admission for alcohol related disease, and trajectories of alcohol consumption over 17 years. In addition, we examined whether cardiometabolic disease modifies the association between alcohol consumption and dementia.

## Methods

### Study population

The Whitehall II study is an ongoing cohort study of men and women originally employed by the British civil service in London based offices.^17^ A total of 10 308 adults (6895 men and 3413 women, aged 35-55) were recruited during 1985-88. Since baseline, follow-up clinical examinations have taken place about every four or five years, with each wave taking two years to complete; the last one was for 2015/16. Written informed consent from participants and research ethical approvals were renewed at each contact.

### Measures

#### Alcohol consumption

Alcohol consumption was assessed over the follow-up period by questionnaire in 1985/88, 1989/90, 1991/93, 1997/99, 2002/04, 2007/09, 2012/13, and 2015/16.

**Fig 1:**
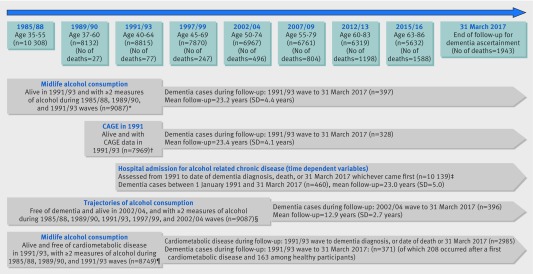
Flow chart of study. Of 10 308 baseline participants, the following were excluded: *1221 (77 died before 1991/93 and 1144 had <2 alcohol measures during 1985/88, 1989/90, and 1991/93 waves); †2339 (77 died before 1991/93 and 2262 did not have data on CAGE or covariates at 1991/93 wave); ‡169 (77 died before 1991/93 and 92 did not have data on covariates during follow-up (missing covariates at a specific wave were replaced by data from closest wave)); §1381 (14 had dementia and 491 died before 2002/04, and 876 had <2 alcohol measures over 1985/88, 1989/90, 1991/93, 1997/99, and 2002/04 waves); ¶1559 (77 died before 1991/93, 1144 had <2 alcohol measures over 1985/88, 1989/90, and 1991/93 waves, and 338 had prevalent cardiometabolic disease)

*Midlife alcohol consumption*—The mean age of participants assessed for alcohol consumption in midlife was 50.3 years (fig 1 and appendix table S1). To reduce measurement error we used the mean of consumption measured in 1985/88, 1989/90, and 1991/93. We converted measures on frequency and number of alcoholic drinks (“measures” of spirits, “glasses” of wine, and “pints” of beer) consumed to units of alcohol consumed per week.

We classified participants who reported no alcohol consumption over the five previous years in 1985/88 and no alcohol consumption in the previous year in 1989/90 and 1991/93 as 10 year abstainers (n=269, 3.0%). Participants who reported alcohol cessation in the previous five years in 1985/88 and those consuming alcohol in 1985/88 or 1989/90 but not in 1991/93 were categorised as former drinkers (n=172, 1.9%). Participants who reported consuming alcoholic beverages in the previous year but not in the last week at all three waves were classified as occasional drinkers (n=862, 9.5%). As these three categories had similar hazards of dementia (see appendix table S2), we combined them into a single category of “abstinence.” Among drinkers, we categorised average alcohol consumption (1985/88 to 1991/93) as 1-14 units/week and >14 units/week to reflect alcohol guidelines in the United Kingdom.^18^

*Alcohol consumption trajectory from midlife to early old age*—We constructed trajectories of alcohol consumption between 1985/88 and 2002/04 (mean age 44.8 and 61.2, respectively; fig 1 and appendix table S1) using our defined categories of alcohol consumption data from 1985/88, 1989/90, 1991/93, 1997/99, and 2002/04 based on 8927 participants who were alive and free of dementia in 2002/04 and who had at least two assessments of alcohol consumption. Trajectories were determined using group based trajectory modelling, fitted using the *traj* command in Stata.^19^ We chose the trajectories based on model fit statistics (bayesian information criterion values and average posterior probabilities) and judgment about whether they adequately addressed the research question (appendix table S3).^20^ The identified trajectories (appendix figure S1) were long term abstinence, decreased alcohol consumption, long term consumption of 1-14 units/week, increased consumption, and long term consumption of >14 units/week.

#### Alcohol dependence

Alcohol dependence was measured by the CAGE questionnaire in 1991/93. This brief four item scale (felt need to Cut down on drinking, Annoyed by people criticising your drinking, Guilty about drinking, Need a drink first thing (Eye-opener)).^21^ It is a validated screening instrument for alcohol dependence that was originally developed for general practice settings and correlates well with a clinical diagnosis of alcoholism. A cut-off threshold of two or more positive responses has been found to identify problems with alcohol.^22^

#### Hospital admission for alcohol related chronic disease

Using the national hospital episode statistics database we identified hospital admissions attributable to alcohol related chronic disease according to the ICD codes (international classification of diseases, ninth and 10th revisions) defined by the Centers for Disease Control and Prevention^23^ (appendix table S4).

#### Dementia

We used comprehensive tracing of electronic health records for dementia ascertainment based on three databases: the national hospital episode statistics, the Mental Health Services Data Set, and the mortality register. Record linkage was until 31 March 2017, using ICD-10 codes F00-F03, F05.1, G30, and G31. The UK National Health Service (England, Scotland, and Wales) provides most of the healthcare, including outpatient and inpatient care; private medical insurance, held by around 12% of the UK population (1997 figures),^24^ is mainly used for elective surgery rather than for chronic conditions such as dementia. The Mental Health Services Data Set is a national database that contains information on people in contact with mental health services in hospitals, outpatient clinics, and the community. Mortality data were drawn from the British national mortality register (National Health Services Central Registry). The tracing exercise was carried out using the unique NHS identification number given to each UK resident. The date of dementia was set at the first record of dementia diagnosis in any of the three databases used for ascertainment.

To assess the validity of the method of dementia ascertainment, we used a mixed model with a backward time scale and determined trajectories of global cognitive score based on performance in three cognitive domains (memory, reasoning, fluency), assessed five times between the 1997/99 and 2015/16 waves.^25^ These results show an accelerated decline in global cognition in the 10 years before the dementia diagnosis (appendix figure S2), as has been shown in studies that use a “gold standard” method for dementia ascertainment.^26^

#### Covariates

Sociodemographic variables included age, sex, ethnicity (white, non-white), marital status (married/cohabiting, other), and socioeconomic status using occupational position (three categories: high, intermediate, and low, representing income and status at work) and education (five categories: less than primary school (age <11), lower secondary school (age <16), higher secondary school (age <18), and university degree and higher).

Health behaviours were assessed over the follow-up period by questionnaire and included smoking (current, former, never), hours of moderate to vigorous physical activity (using questions on moderately energetic (eg, dancing, cycling, leisurely swimming) and vigorous (eg, running, hard swimming, playing squash) physical activity at baseline and then using a modified version of the Minnesota leisure time physical activity questionnaire),^25^ and dietary behaviour (frequency of fruit and vegetables consumed in a week).

Health related covariates included systolic blood pressure, total cholesterol level, prevalent diabetes mellitus (determined by fasting blood glucose ≥7.0 mmol/L, reported doctor diagnosed diabetes, use of antidiabetic drugs, or hospital records), body mass index (categorised as <20, 20-24.9, 25-29.9, and ≥30 kg/m^2^) assessed by a trained nurse at the clinical examination through blood tests and standard clinical protocols, cardiovascular diseases (including coronary heart disease, stroke, atrial fibrillation, and heart failure) identified using linkage to national hospital records, self reported use of drugs for cardiovascular disease, and the 30 item general health questionnaire on anxiety and depression symptoms.^27^

### Statistical analysis

Three sets of analyses were undertaken (fig 1 and appendix table S1). Cox regression was used in all analyses, with age as the timescale to model the associations with hazard of incident dementia. Participants were censored at date of record of dementia, death, or 31 March 2017, whichever came first. Models were first adjusted for sociodemographic factors, then additionally for health behaviours, and finally for health status.

#### Analysis of midlife alcohol consumption

We examined the association of alcohol consumption in midlife (mean of 1985/88, 1989/90, and 1991/93), alcohol dependence in 1991/93, and hospital admission for alcohol related chronic diseases from 1991, with follow-up for dementia starting in 1991. Covariates were assessed in 1991/93 (or the closest wave if missing), apart for the analysis on hospital admission for alcohol related chronic diseases, for which all covariates were time varying. We first examined the shape of the association of midlife alcohol consumption and dementia using restricted cubic spline regressions with Harrell knots,^28^ and Stata command xblc^29^ with 14 units/week consumption as the reference. In subsequent Cox regression we assessed the association of alcohol consumption categories (abstinence, 1-14 units/week, and >14 units/week) with risk of dementia. In those reporting consumption of >14 units/week, we used spline regressions to examine whether there was a linear trend in the association of alcohol consumption with dementia and then estimated the risk of dementia for every increment of 7 units/week.

#### Trajectories of alcohol consumption from midlife to early old age

The aim of these analyses was to examine the association of long term alcohol consumption with risk of dementia using trajectories of alcohol consumption between 1985/88 and 2002/04 waves; the follow-up started in 2002/04 and covariates were drawn from the 2002/04 wave (or the closest wave if missing).

#### Role of cardiometabolic disease in association between alcohol consumption and dementia

In subsequent analyses we examined the mediating role of cardiometabolic disease (stroke, coronary heart disease, atrial fibrillation, heart failure, and diabetes) over the follow-up period in the association between alcohol consumption and risk of dementia. These analyses were carried out using multistate models (package mstate, R software) in participants free of dementia and cardiometabolic disease in 1991/93. The models allow simultaneous estimation of the risk associated with alcohol consumption in three transitions: from healthy state to incident cardiometabolic disease, from cardiometabolic disease to incident dementia, and from healthy state to incident dementia in those free of cardiometabolic disease. Age was used as the timescale, and the analyses were adjusted for sociodemographic factors.

#### Sensitivity analysis

Five sets of sensitivity analyses were undertaken. Firstly, we examined the association between alcohol consumption in midlife and risk of mortality in the total sample using Cox regression and then in those without a diagnosis of dementia during the follow-up period using multistate models. We used the modified Fine and Gray competing risks method to address the possibility that those who died could have developed dementia had they lived longer.^30^ Secondly, we repeated analysis on the shape of the association between alcohol consumption and risk of dementia excluding abstainers in order to check for potential distortion of the association due to abstainers. Thirdly, to examine whether alcohol consumption at age 50, 60, and 70 was similarly associated with risk of dementia, we extracted data on alcohol consumption at these ages for each participant across the data waves, allowing a five year margin either way for each age category, and we assessed the association with incident dementia using Cox models; start of follow-up and covariates were from age 50, 60, and 70, respectively. Fourthly, we examined the associations of type of alcohol consumed using mean midlife consumption (1985/88, 1989/90, and 1991/93) using restricted cubic splines adjusted for sociodemographic variables and mutually adjusted for types of alcohol consumed. Finally, to examine whether poor sensitivity of our method of dementia ascertainment biased our results, we simulated scenarios with differential misclassification (ie, the hypothesis that proportion of misclassified dementia cases depends on midlife alcohol consumption categories) using a SAS macro provided by Fox et al.^31^ We allowed the sensitivity to range between 60% and 90% and the specificity between 97% and 100% using a trapezoidal probability density function. We first analysed the impact of potential differential misclassification on the hazard ratio estimate associated with abstinence compared with alcohol consumption of 1-14 units/week. To account for differential misclassification, we used separate sensitivity (specificity) distributions in the two alcohol consumption groups (abstinence and 1-14 units/week). We simulated two scenarios: first where the correlation of the two sensitivity (specificity) distributions in the alcohol consumption groups was 0.8 and then 0.6 (a correlation of 1 would correspond to non-differential misclassification). We repeated these analyses to assess the impact of differential misclassification on the comparison between alcohol consumption of >14 units/week compared with 1-14 units/week. Cox regression analyses were undertaken using STATA 15, multistate models using R, and sensitivity analyses on dementia misclassification using SAS 9.4.

### Patient involvement

Participants of the Whitehall II study were not involved in setting the research question or the outcome measures, nor were they involved in developing plans for recruitment, design, or implementation of the study. No participants were asked advice on interpretation or writing up of results. However, all results are disseminated to study participants through newsletters and our website, which has a participant portal, www.ucl.ac.uk/whitehallII/participants/.

## Results

Among the 10 231 participants alive in 1991/93, 9087 had at least two measurements of alcohol consumption between 1985/88 and 1991/93 and complete data on covariates (fig 1). Among these participants, a total of 397 cases of dementia were recorded over a mean follow-up of 23.2 (SD 4.4, range 0.08-25.6) years. Mean age at dementia diagnosis was 75.6 (SD 5.8; interquartile range 72.2-80.0; range 53.4-85.9) years, with the first case recorded in 1995 and 72% (287 cases out of 397) of the cases recorded in the last five years of follow-up. Greater age (hazard ratio 1.21 per 1 year older age at study baseline, 95% confidence interval 1.18 to 1.23), female sex (1.57, 1.29 to 1.92), education less than secondary school diploma (1.68, 1.38 to 2.05), and low occupational position (2.39, 1.94 to 2.95) were associated with a greater hazard of dementia. Table 1 presents the characteristics of the study population (n=9087). Abstainers were more likely to be women, non-white, and from the lower socioeconomic group. They also had a worse cardiometabolic profile (table 1). Participants in the 1-14 units/week alcohol consumption group were more likely to drink wine and those in the >14 units/week group to drink beer (table 1).

**Table 1:** Characteristics of study population in 1991/93.* Values are numbers (percentages) unless stated otherwise

Characteristics	Dementia status at end of follow-up				Average alcohol consumption during 1985/88, 1989/90, and 1991/93 waves			
	No dementia	Dementia	P value		Abstinence	1-14 units/week	>14 units/week	P value
No of participants	8690	397			1303	5552	2232	
Mean (SD) age (years)	50.0 (6.0)	55.8 (4.7)	<0.001		51.4 (6.1)	50.4 (6.2)	49.2 (5.8)	<0.001
Women	2708 (31.2)	166 (41.8)	<0.001		717 (55.0)	1887 (34.0)	270 (12.1)	<0.001
Non-white	789 (9.1)	53 (13.4)	0.004		341 (26.2)	427 (7.7)	74 (3.3)	<0.001
Less than secondary school diploma	4016 (46.2)	232 (58.4)	<0.001		783 (60.1)	2658 (47.9)	807 (36.2)	<0.001
Low occupational position	1521 (17.5)	131 (33.0)	<0.001		550 (42.2)	961 (17.3)	141 (6.3)	<0.001
Married/cohabiting	6606 (76.0)	293 (73.8)	0.31		865 (66.4)	4296 (77.4)	1738 (77.9)	<0.001
Units/week:								
Alcohol	10.3 (12.0)	8.4 (11.8)	0.002		0 (0)	6.0 (3.7)	26.5 (13.4)	<0.001
Wine	3.7 (4.8)	2.8 (4.3)	<0.001		0 (0)	2.8 (2.5)	7.9 (7.1)	<0.001
Beer	4.7 (8.8)	3.6 (8.8)	0.02		0 (0)	2.0 (2.6)	13.7 (7.9)	<0.001
Spirit	2.0 (3.9)	2.0 (4.5)	0.98		0 (0)	1.2 (1.7)	4.9 (6.6)	<0.001
CAGE cases^†^	780 (10.2)	33 (10.1)	0.93		38 (4.4)	253 (5.0)	522 (25.4)	<0.001
≥1 hospital admission for alcohol related disease**^‡^**	138 (1.6)	15 (3.8)	0.001		4 (0.3)	37 (0.7)	112 (5.0)	<0.001
Current smokers	1307 (15.0)	74 (18.6)	0.05		201 (15.4)	764 (13.7)	416 (18.7)	<0.001
Mean (SD) moderate to vigorous physical activity (hrs)	3.9 (4.3)	3.5 (3.8)	0.04		3.1 (4.5)	3.9 (4.2)	4.4 (4.2)	<0.001
Daily fruit and vegetable consumption	5302 (61.0)	231 (58.2)	0.26		770 (59.1)	3486 (62.8)	1277 (57.2)	<0.001
Body mass index ≥30	813 (9.4)	60 (15.1)	0.001		186 (14.3)	504 (9.1)	183 (8.2)	<0.001
Diabetes	202 (2.3)	20 (5.0)	0.001		59 (4.5)	111 (2.0)	52 (2.3)	<0.001
Mean (SD) total cholesterol (mmol/L)	6.4 (1.2)	6.7 (1.3)	<0.001		6.4 (1.2)	6.4 (1.2)	6.6 (1.2)	<0.001
Mean (SD) systolic blood pressure (mm Hg)	121.0 (14.0)	124.3 (15.2)	<0.001		121.2 (14.8)	120.2 (13.8)	123.4 (13.9)	<0.001
Cardiovascular disease	187 (2.2)	17 (4.3)	0.005		38 (2.9)	112 (2.0)	54 (2.4)	0.12
Cardiovascular disease drugs	705 (8.1)	75 (18.9)	<0.001		157 (12.1)	451 (8.1)	172 (7.7)	<0.001
General health questionnaire score	3.1 (5.2)	3.2 (5.3)	0.66		3.1 (5.5)	3.0 (5.0)	3.3 (5.5)	0.05

* All data are drawn from 1991/93, baseline of study population.

^†^ Based on those with available data (n=7969, number of cases=328), cases defined as CAGE score ≥2.

^‡^ Numbers differ with those in table 2 as values are for those with available data on five year alcohol consumption.

### Midlife alcohol consumption

Age at dementia diagnosis was 76.1, 75.7, and 74.4 years (P=0.13) in the abstinence, 1-14 units/week, and >14 units/week groups, respectively. As no evidence was found of an interaction between alcohol consumption and age (P=0.76), or sex (P=0.92), or occupational position (P=0.95) in associations with dementia, we combined these subgroups in the analyses.

**Fig 2:**
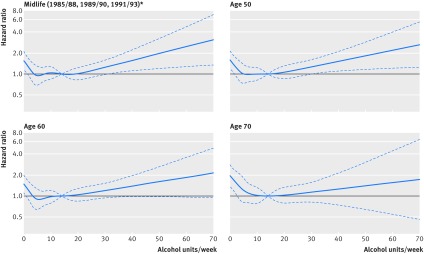
Association between alcohol consumption per week and risk of dementia by age. *Cox regression analysis adjusted for sociodemographic factors. Dotted lines represent 95% confidence intervals

Figure 2 shows the association between alcohol consumption in midlife and risk of dementia in analysis adjusted for sociodemographic factors; age at which alcohol consumption was assessed did not modify this association (fig 2 and appendix table S5). Abstinence was associated with a higher risk of dementia when the reference was alcohol consumption of 14 units/week; in these analyses alcohol consumption >14 units/week was associated with an increased risk of dementia in a linear fashion (among those drinking >14 units/week, P for non-linearity=0.97 using spline regressions). In a model adjusted for sociodemographic factors alcohol abstinence was associated with a greater risk of dementia (hazard ratio 1.47, 1.15 to 1.89) compared with alcohol consumption of 1-14 units/week (table 2). Among those drinking >14 units/week, a 7 unit increase in alcohol consumption was associated with a 17% (95% confidence interval 4% to 32%) increase in risk of dementia. Additional adjustment for health behaviours and health related variables did not attenuate the observed associations. Use of time varying health related factors (data not shown) did not change the results: the hazard ratio for abstainers was 1.44 (1.11 to 1.85) and for each 7 unit increase in consumption among those drinking >14 units/week was 1.18 (1.05 to 1.34).

**Table 2:** Association between alcohol consumption and risk of dementia

Variables	No of cases/total No	Hazard ratio (95% CI)		
		Adjusted for sociodemographic variables^†^	Additionally adjusted for behavioural factors^‡^	Fully adjusted^§^
**Average alcohol consumption in midlife (1985/88, 1989/90, and 1991/93)^¶^; cases=397/9087; mean follow-up 23.2 (SD 4.4) years**				
Abstinence	98/1303	1.47 (1.15 to 1.89)*	1.48 (1.15 to 1.91)*	1.45 (1.12 to 1.86)*
1-14 units/week	229/5552	1 (ref)	1 (ref)	1 (ref)
>14 units/week	70/2232	1.08 (0.82 to 1.43)	1.05 (0.80 to 1.39)	1.02 (0.77 to 1.35)
**Among those drinking >14 units/week**				
Per 7 units/week increase	70/2232	1.17 (1.04 to 1.32)*	1.19 (1.05 to 1.34)*	1.18 (1.04 to 1.34)*
CAGE score in 1991/93^**^: cases=328/7969; mean follow-up 23.4 (SD 4.1) years				
0	253/5727	1 (ref)	1 (ref)	1 (ref)
1	42/1429	0.93 (0.67 to 1.29)	0.93 (0.66 to 1.30)	0.90 (0.64 to 1.26)
2	18/574	1.03 (0.64 to 1.66)	1.01 (0.62 to 1.63)	0.96 (0.59 to 1.56)
3/4	15/239	2.19 (1.29 to 3.71)*	2.13 (1.25 to 3.61)*	1.98 (1.15 to 3.38)*
**CAGE caseness**				
No (0/1)	295/7156	1 (ref)	1 (ref)	1 (ref)
Yes (≥2)	33/813	1.37 (0.95 to 1.97)	1.34 (0.93 to 1.93)	1.27 (0.88 to 1.84)
**Hospital admission for alcohol related disease from 1991††; cases=460/10 139; mean follow-up 23.0 (SD 5.0) years**				
None	440/9946	1 (ref)	1 (ref)	1 (ref)
At least one during follow-up	20/193	4.28 (2.72 to 6.73)*	3.70 (2.34 to 5.86)*	2.95 (1.85 to 4.71)*

* P<0.05.

† Adjusted for age (time scale), sex, ethnicity, education, occupational position, and marital status.

‡ Additionally adjusted for physical activity, smoking status, and fruit and vegetable consumption.

§ Additionally adjusted for systolic blood pressure, total cholesterol, diabetes, body mass index, general health questionnaire score, cardiovascular disease, and cardiovascular disease drugs.

¶ Participants with at least two measures between 1985/88 and 1991/93 (78.8% had three measures and 21.2% had two measures).

** CAGE was used for first time in 1991/93.

†† All data entered as time varying covariates.

Compared to those with a CAGE score of 0, a higher risk of dementia was observed in those with a CAGE score >2 (hazard ratio 2.19, 1.29 to 3.71). This association was slightly attenuated in the fully adjusted model (table 2). In analysis adjusted for sociodemographic factors, one hospital admission or more for alcohol related chronic disease over follow-up was associated with a 4.3 times higher risk of dementia (95% confidence interval 2.7 to 6.7). This hazard ratio reduced to 3.0 (1.9 to 4.7) in fully adjusted analysis (table 2).

### Trajectories of alcohol consumption between midlife and early old age

We identified five trajectories of alcohol consumption (appendix figure S1): long term abstinence (9% of participants were in this group), decreased consumption (6%), long term consumption 1-14 units/week group (59%), increased consumption (11%), and long term consumption >14 units/week (14%). Compared with participants in the long term consumption 1-14 units/week group, those with long term abstinence (1.74, 1.31 to 2.30), decreased consumption (1.55, 1.08 to 2.22), and long term consumption >14 units/week (1.40, 1.02 to 1.93) had a higher risk of dementia. These associations remained after adjustment for behavioural and health related factors (table 3).

**Table 3:** Association of alcohol consumption trajectories between 1985/88 and 2002/04 with risk of dementia

**Alcohol consumption trajectories**	No of cases/total No	Hazard ratio (95% CI)		
		Adjusted for socio-demographic variables^†^	Additionally adjusted for behavioural factors^‡^	Fully adjusted^§^
**Cases=396/8927, mean follow-up 12.9 (SD 2.7) years**				
Long term abstinence	74/837	1.74 (1.31 to 2.30)*	1.73 (1.30 to 2.29)*	1.67 (1.26 to 2.23)*
Decreased consumption	36/500	1.55 (1.08 to 2.22)*	1.53 (1.07 to 2.20)*	1.50 (1.04 to 2.16)*
Long term consumption 1-14 units/week	207/5304	1 (ref)	1 (ref)	1 (ref)
Increased consumption	28/1004	0.88 (0.59 to 1.31)	0.87 (0.59 to 1.30)	0.85 (0.57 to 1.26)
Long term consumption >14 units/week	51/1282	1.40 (1.02 to 1.93)*	1.39 (1.01 to 1.92)*	1.36 (0.99 to 1.88)

* P<0.05.

^†^ Adjusted for age (time-scale), sex, ethnicity, education, occupational position, and marital status.

^‡^ Additionally adjusted for physical activity, smoking status, and fruit and vegetable consumption.

^§^ Additionally adjusted for systolic blood pressure, total cholesterol, diabetes, body mass index, general health questionnaire score, cardiovascular disease, and cardiovascular disease drugs.

### Role of cardiometabolic disease in association between midlife alcohol consumption and dementia

**Fig 3:**
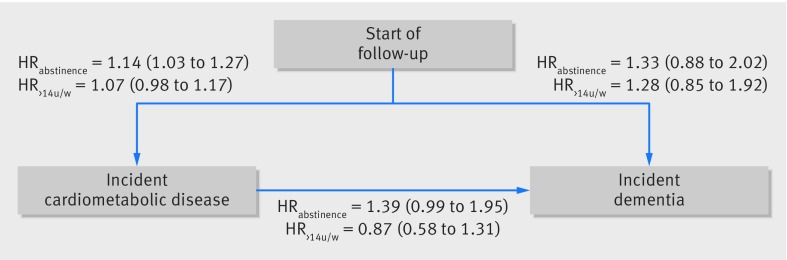
Multistate models for role of midlife alcohol consumption in transitions to cardiometabolic disease and dementia in all participants.* Analysis based on 8749 participants free of cardiometabolic disease and dementia in 1991/93; 2985 participants had incident cardiometabolic disease (among whom 208 developed dementia); among healthy participants 163 had dementia. Analysis adjusted for age, sex, ethnicity, education, occupational position, and marital status. HR_abstinence_=hazard ratio for abstainers versus alcohol consumption of 1-14 units/week; HR_>14u/w_=hazard ratio for >14 units/week versus alcohol consumption o=1-14 units/week

**Fig 4:**
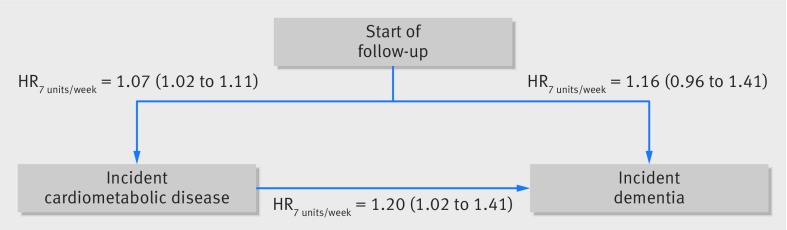
Multistate models for role of midlife alcohol consumption in transitions to cardiometabolic disease and dementia in participants with midlife alcohol consumption >14 units/week. Analysis based on 2143 participants free of cardiometabolic disease and dementia in 1991/93; 709 participants had incident cardiometabolic disease (among whom 34 developed dementia); among healthy participants 32 had dementia. Analysis adjusted for age, sex, ethnicity, education, occupational position, and marital status. *HR_7 units/week_* associated with a 7 unit increase in alcohol consumption

The mediating role of cardiometabolic disease was examined using multistate models (figs 3 and 4). Among those without cardiometabolic disease, compared with alcohol consumption of 1-14 units/week, the hazard ratio for dementia associated with abstinence was 1.33 (0.88 to 2.02) and with consumption >14 units/week was 1.28 (0.85 to 1.92) (fig 3); corresponding hazard ratios in the entire population, when cardiometabolic disease was not taken into consideration, were 1.47 (1.15 to 1.89) and 1.08 (0.82 to 1.43; table 2). In participants consuming >14 units/week (fig 4), cardiometabolic disease did not seem to play a role, as the hazard ratio associated with each increase of 7 units/week was 1.16 (0.96 to 1.41), similar to that observed when cardiometabolic diseases were not taken into account (1.17, 1.04 to 1.32; table 2).

### Sensitivity analysis

Analysis using Cox regression showed that compared with alcohol consumption of 1-14 units/week, the hazard ratio for mortality over follow-up for abstinence was 1.15 (0.99 to 1.32) and for alcohol consumption >14 units/week was 1.31 (1.15 to 1.48; appendix table S6). Multistate models (appendix figure S3) showed that these hazard ratios among participants without a dementia diagnosis during follow-up were reduced to 0.97 (0.67 to 1.41) and 1.10 (0.74 to 1.66), respectively, and the risk of mortality increased by 13% (7% to 19%) for each 7 unit increment among participants consuming >14 units/week.

Results for dementia from the modified Fine and Gray model that accounts for competing risks of mortality were similar to those in the main analysis (table 4). Appendix figure S4 shows the shape of the association between alcohol consumption in midlife and risk of dementia to be unaffected by the exclusion of the large group of abstainers from the analysis. Regardless of type of alcohol consumed, the risk of dementia increased linearly, starting around 14 units/week (appendix figure S5). Subsidiary analyses examining potential bias due to differential misclassification of dementia suggested our main findings on the association of alcohol consumption with dementia to be robust (appendix table S7).

**Table 4:** Association between midlife alcohol consumption and risk of dementia in competing risk analysis^†^

Variables	Dementia cases/total	Hazard ratio (95% CI)	
		Cox regression	Competing risk analysis
**Average alcohol consumption in midlife (1985/88, 1989/90, and 1991/93)**			
Abstinence	98/1303	1.47 (1.15 to 1.89)*	1.44 (1.21 to 1.71)*
1-14 units/week	229/5552	1 (ref)	1 (ref)
>14 units/week	70/2232	1.08 (0.82 to 1.43)	1.09 (0.91 to 1.30)
**Among those drinking >14 units/week**			
Per 7 units/week increase	70/2232	1.17 (1.04 to 1.32)*	1.16 (1.07 to 1.25)*

* P<0.05.

^†^ Modified Fine and Gray competing risk analysis.^30^ Cases=397/9087; mean follow-up=23.2 (SD 4.4) years. Adjusted for age (time scale), sex, ethnicity, education, occupational position, and marital status.

## Discussion

In this longitudinal study, multiple approaches to examine the association between alcohol consumption and dementia present converging evidence on three key findings on abstinence, excessive alcohol consumption, and the role of cardiometabolic disease. First, the risk of dementia was higher in those abstaining from alcohol in midlife. Alcohol consumption trajectories from midlife to early old age supported these findings—both long term abstainers and those reporting decreased alcohol consumption had an increased risk of dementia. Second, alcohol consumption >14 units/week increased the risk of dementia in a linear fashion; an excess risk that was evident when alcohol consumption was assessed at ages 50, 60, and 70 years. Data using hospital admission for chronic disease caused by high alcohol consumption showed a four times higher risk of dementia, supporting findings on the neurotoxic effects of alcohol consumption >14 units/week. Thirdly, multistate models showed that part of the excess risk of dementia in abstainers was attributable to the greater risk of cardiometabolic disease in this group. Taken together, these results suggest that abstention and excessive alcohol consumption are associated with an increased risk of dementia, although the underlying mechanisms are likely to be different in the two groups. Overall, no evidence was found that alcohol consumption between 1 unit/week and 14 units/week increases the risk of dementia.

### Strengths and limitations of this study

The present study has several strengths. Repeat assessment of alcohol consumption allowed us to assess mean midlife alcohol consumption in order to minimise biases due to measurement error, examine associations with dementia of trajectories of alcohol consumption between midlife and early old age, and examine whether age modifies associations between alcohol consumption and dementia. These features, along with a mean follow-up period of 23 years, allowed a comprehensive assessment of the association of alcohol consumption with dementia. Besides measurement error, studies that recruit participants at older ages are not able to assess the excess risk in those who change their alcohol consumption with age. We were also able to examine the shape of the association between alcohol consumption >14 units/week and dementia, which was similar to that reported in a recent meta-analysis.^7^ Dose-response assessment by meta-analysis can be problematic for heavy alcohol consumption as the estimate is constrained to the mean or median consumption in the high alcohol consumption category.^7^ Finally, we used multistate models to examine the role of cardiometabolic disease and we undertook further analyses to take the competing risk of mortality into account where results were similar to those obtained using Cox regression, increasing confidence in our main findings.

The study findings need to be interpreted keeping in mind the observational nature of the data. A key limitation, as in other observational studies, is the measurement of alcohol consumption using self reports. It is possible that systematic reporting biases affected findings, although comparison of alcohol consumption reported by the participants of the Whitehall II study suggests patterns similar to those in several other UK cohort studies.^11^ The use of multiple approaches, including the measures of hospital admission for alcohol related chronic disease with converging findings, suggest that our results on excessive alcohol consumption are robust.

The ascertainment of dementia based on linkage to electronic health records has advantages and disadvantages. A recent study^32^ reported that passive assessment of dementia through UK hospital records has high specificity but modest sensitivity (78%) owing to milder cases of dementia being missing, as also found in the Mayo Clinic Study of Aging and the Adult Changes study.^33^ In addition to hospital records, we used other sources of dementia diagnosis, such as the UK mental health database, which is likely to improve the sensitivity of dementia diagnosis. Accordingly, our analyses, which simulated differential misclassification scenarios, show the results to be robust. Our findings are also in accordance with previous findings from the Whitehall II study suggesting that both alcohol abstinence and high alcohol consumption are associated with accelerated cognitive decline.^34^ The advantage of ascertainment through linkage to health records is that it allows analysis of everyone recruited to the study rather than only those who continue to participate in the study over a long follow-up and are available for an in-person ascertainment of dementia. A further difficulty of face-to-face assessment is that people can develop dementia and die between two assessments, which prevents them from being categorised as having dementia. Such bias is particularly likely with risk factors that also affect mortality, as is the case with excessive alcohol consumption.

### Comparison with other studies

A recent meta-analysis of observational studies concluded that light to moderate alcohol consumption is associated with a reduced risk of dementia, whereas both abstinence and heavy drinking are associated with a higher risk of dementia.^7^ In seven of the 10 studies included in the analysis, the mean follow-up period was less than 10 years, with alcohol consumption being assessed later in life and thus potentially modified by health related problems.^11^ In two of the three other studies, the CAIDE^35^ and the Finnish Twin^36^ cohorts, although a U-shaped association was suggested, results were not robust owing to the small number of cases in the extreme alcohol consumption categories. In a study based on the Swedish Twin Registry with 43 years of follow-up, where dementia was assessed through electronic health records, a quadratic association was found whereby both no alcohol consumption and high alcohol consumption were associated with an increased risk of dementia, although the excess risk in abstainers did not reach statistical significance and the excess risk of high consumption began at 12 g/day (corresponding to around 10.5 units/week).^37^ The study also found, as in our investigation, a reduced risk of dementia for moderate wine consumption and a linear increased risk of dementia in those consuming spirits. Furthermore, a recent study based on a nationwide dataset of patients admitted to hospital in France between 2008 and 2013 reported that those with a hospital admission record for alcohol use disorders had a 3.3-times higher risk of dementia in multivariate analysis,^12^ providing further support for our findings.

We, as with others,^7^ observed an increased risk of dementia in alcohol abstainers, a finding subject to much debate. As studies usually assess alcohol consumption only once, excess risk might be driven by the inclusion of former drinkers in the same group as abstainers.^7^ Our analyses using repeat data on alcohol consumption across midlife suggest that former drinking might not explain the excess dementia risk in abstainers, although we cannot exclude the possibility that those who report alcohol abstinence in midlife were heavy drinkers in young adulthood or misreported their alcohol consumption. We accounted for several sociodemographic and health related characteristics in the analysis, but residual confounding cannot be excluded as an explanation for the higher risk of dementia among abstainers. Indeed, this group is particular in that it is composed mainly of women from the lower socioeconomic group with higher prevalence of cardiometabolic risk factors and disease at baseline, a pattern that has also been observed in other studies.^35^ ^37^

Alcohol abstinence is also associated with a higher risk of diabetes and cardiovascular disease,^1^ ^15^ which might increase the risk of dementia.^4^ ^16^ A recent study of nearly 600 000 people, for example, found a J-shaped association between alcohol consumption and cardiovascular disease, with a weekly alcohol consumption of 100 g (12.5 units) being associated with the lowest risk of cardiovascular disease and a higher disease risk observed in those consuming smaller amounts of alcohol.^14^ Moderate alcohol consumption has been hypothesised to benefit cardiovascular health through favourable impacts on lipid profile, inflammation level, endothelial function, and insulin sensitivity.^13^ ^38^ In agreement with this, a meta-analysis of interventional studies (alcohol use versus a period of no alcohol use) reported that moderate alcohol consumption had favourable effects on levels of high density lipoprotein cholesterol, apolipoprotein A1, adiponectin, and fibrinogen,^38^ potentially underlying the apparent neuroprotective effects of moderate alcohol consumption. Our multistate models lent partial support for a mediating role of cardiometabolic disease in the association between alcohol abstention and increased risk of dementia; the hazard ratio of dementia associated with alcohol abstinence, compared with moderate consumption, was reduced to 1.33 (95% confidence interval 0.88 to 2.02) in those without cardiometabolic disease, compared with 1.47 (1.15 to 1.89) in the entire population. Nevertheless, studies using other approaches such as mendelian randomisation^39^ and brain imaging data from a subsample of the Whitehall II study^40^ suggest linear adverse effects of alcohol consumption. The present findings on alcohol abstinence should therefore not motivate people who do not drink to start drinking given the known detrimental effects of alcohol consumption for all cause mortality and diseases such as neuropsychiatric disorders, cirrhosis of the liver, and cancer.^1^

Excessive alcohol drinking is detrimental for the brain,^6^ ^7^ ^41^ ^42^ but the level from which this effect is evident is less clear. We assessed the impact of alcohol consumption using the 14 units/week threshold advocated by guidelines in the United Kingdom.^18^ From this level onwards, the risk of dementia increased in a linear fashion. In addition, long term exposure to alcohol consumption above this limit increased the risk of dementia by 50% compared with long term moderate consumption (1-14 units/week). These results support the recent downward revision of UK guidelines that moved the recommended alcohol consumption limit to 14 units/week in men compared with 21 units/week before, bringing them in line with women. Analyses on alcohol dependence scale and hospital admission for alcohol related chronic disease strengthen the evidence that excessive alcohol consumption is a risk factor for dementia. The negative impact of heavy alcohol intake on the risk of dementia has been suggested to involve nutritional deficiency,^42^ ^43^ the direct neurotoxic effects of ethanol,^42^ and the indirect negative impacts through increased risk of diabetes,^44^ hypertension,^45^ and stroke.^46^ ^47^

### Conclusion

Given the number of people living with dementia is expected to triple by 2050^3^ and the absence of a cure, prevention is key.^4^ We show that both long term alcohol abstinence and excessive alcohol consumption may increase the risk of dementia. The UK guidelines suggest an alcohol threshold of 14 units/week but many countries use a much higher threshold to define excessive consumption.^48^ The present study encourages the use of a lower threshold of alcohol consumption in such guidelines, applicable over the adult life course, in order to promote cognitive health.

**What is already known on this topic**

Moderate alcohol consumption is thought to be associated with a lower risk of dementia; the association of alcohol with cognitive outcomes appears to be J-shaped or U-shaped, with harmful effects of both abstinence and excessive alcohol consumption
The evidence is far from robust, however, as excessive alcohol consumption is not included in current guidelines to prevent or delay dementia onset
Inconsistency in findings stems from the fact that most studies assess alcohol consumption in late life, which may not reflect lifetime consumption, and selection bias is likely to affect these findings as studies used face-to-face assessment of cognitive status

**What this study adds**

The results show a greater risk of dementia in those who abstain from alcohol or consume >14 units/week, with risk increasing in a linear fashion at higher levels of consumption
Data on hospital admission for chronic disease caused by high alcohol consumption showed a fourfold higher risk of dementia in these people
The study also found support for a mediating role of cardiometabolic disease; some of the excess risk of dementia in abstainers was explained by greater risk of cardiometabolic disease in this group

## Web extra

Extra material supplied by authors

## Notes

Contributors: ASM and SS developed the hypothesis and study design. SS, AF, and AD performed the statistical analysis. SS wrote the first and successive drafts of the manuscript. All authors conceived and designed the study, analysed and interpreted the data, and drafted or critically revised the manuscript for important intellectual content, or, in addition, acquired data. ASM and MK obtained funding for the Whitehall II study. SS, AD, and ASM had full access to the data and take responsibility for the integrity of the data and the accuracy of the data analysis. SS is the guarantor. The corresponding author attests that all listed authors meet authorship criteria and that no others meeting the criteria have been omitted.

Ethical approval: This study was approved by University College London Hospital Committee on the Ethics of Human Research (reference No 85/0938). Written informed consent from participants and research ethics approvals were renewed at each contact.

Data sharing: Data, protocols, and other metadata of the Whitehall II study are available to the scientific community. Please refer to the Whitehall II study data sharing policy at www.ucl.ac.uk/whitehallII/data-sharing.

Transparency: The lead author (SS) affirms that this manuscript is an honest, accurate, and transparent account of the study being reported; that no important aspects of the study have been omitted; and that any discrepancies from the study as planned have been explained.
