# Supplementary material for: Alcohol consumption and risk of dementia: 23 year follow-up of Whitehall II cohort study
Source: BMJ. 2018 Aug 1;362:k2927. doi: 10.1136/bmj.k2927 (PMC6066998; doi:10.1136/bmj.k2927)
Supplement: Supplementary file 1 — Supplementary information: figures S1-S5 and tables S1-S7 [file sabs044416.ww1.pdf]

Alcohol consumption and risk of dementia: a 23-year follow-up of the Whitehall II cohort study

Séverine Sabia, *Research Associate**[1,2]

Aurore Fayosse, *Statistician*[1]

Julien Dumurgier, Associate Professor [3]

Aline Dugravot, *Statistician*[1]

Tasnime Akbaraly, *Research Associate*[2,4,5]

Annie Britton, *Professor* [2]

Mika Kivimäki, *Professor* [2]

Archana Singh-Manoux, *Research Professor* [1,2]

[1] Inserm, U1018

Centre for Research in Epidemiology and Population Health, Université Paris-Saclay, France

Hôpital Paul Brousse, Bât 15/16, 16 Avenue Paul Vaillant Couturier

94807 VILLEJUIF CEDEX, France

Telephone: +33 (0)1 45 59 51 52

Email: severine.sabia@inserm.fr

[2] Department of Epidemiology and Public Health, University College London, UK

[3] Cognitive Neurology Center, Lariboisière – Fernand Widal hospital, AP-HP, Université Paris Diderot, Sorbonne Paris Cité; Paris, France.

[4] Inserm U 1198, Montpellier F-34000, France. University Montpellier, Montpellier, F-34000, France. EPHE, Paris, France.

[5] Department of Psychiatry & Autism Resources Centre, University Research and Hospital Center (CHRU) of Montpellier, F-34000, Montpellier, France

Online Only Supplements

Figure S1. Trajectories of alcohol consumption between 1985/88 and 2002/04.

Figure S2. Trajectories of cognitive function before dementia diagnosis.

Figure S3. Role of midlife alcohol consumption in transitions to dementia and mortality: a multi-state model approach.

Figure S4. Association between midlife alcohol consumption and incidence of dementia among drinkers* (A) and distribution of midlife alcohol consumption among drinkers (B).

Figure S5. Association between midlife alcohol consumption and dementia by type of alcohol (left panel) and corresponding distribution of types of alcohol consumed (right panel).

Table S1. Mortality and dementia incidence rates over time.

Table S2. Association between detailed categories of midlife alcohol consumption with incident dementia.

Table S3. Estimation of trajectories of alcohol consumption: model fit statistics (group based trajectory models).

Table S4. ICD codes for identification of chronic diseases 100% attributable to alcohol consumption.

Table S5. Association between alcohol consumption at age 50, 60, and 70 and risk of dementia up to March 31st, 2017.

Table S6. Association between midlife alcohol consumption and risk of mortality.

Table S7. Sensitivity analysis to assess the impact of dementia misclassification on the association between alcohol consumption in midlife and dementia.

Figure S1. Trajectories* of alcohol consumption between 1985/88 and 2002/04.

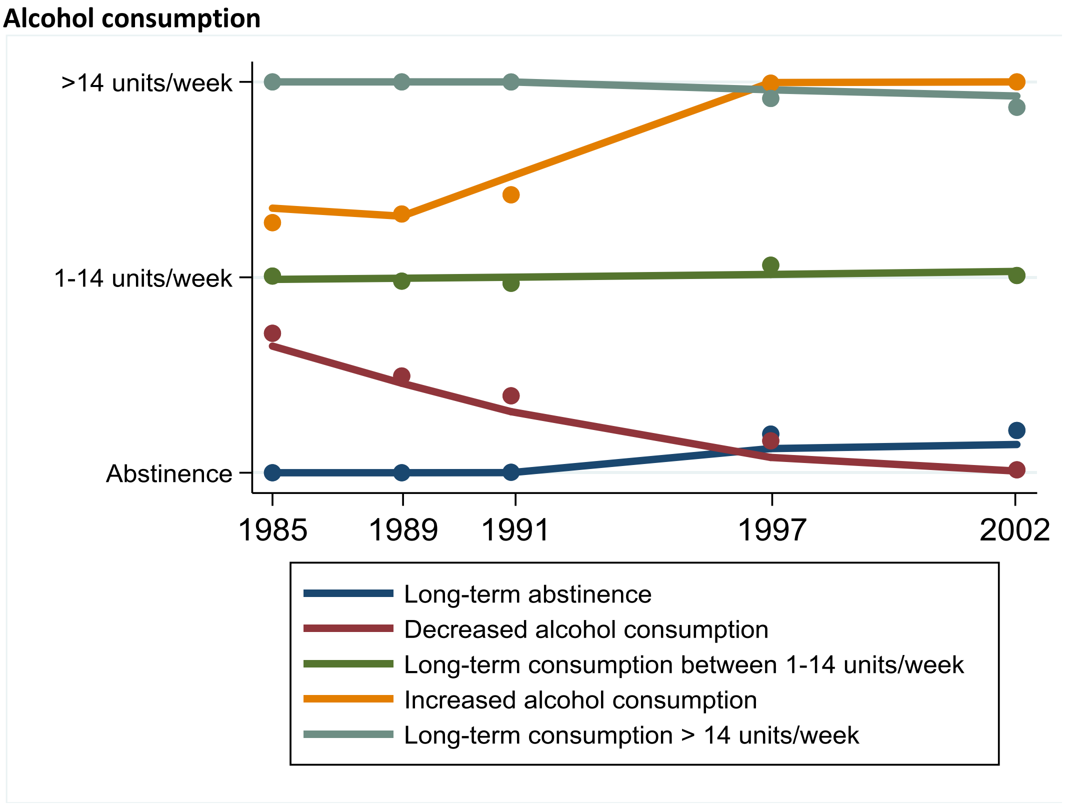

*9% with 2 measures, 12% with 3 measures, 20% with 4 measures, 59% with 5 measures.

Figure S2. Trajectories of cognitive function before dementia diagnosis.*

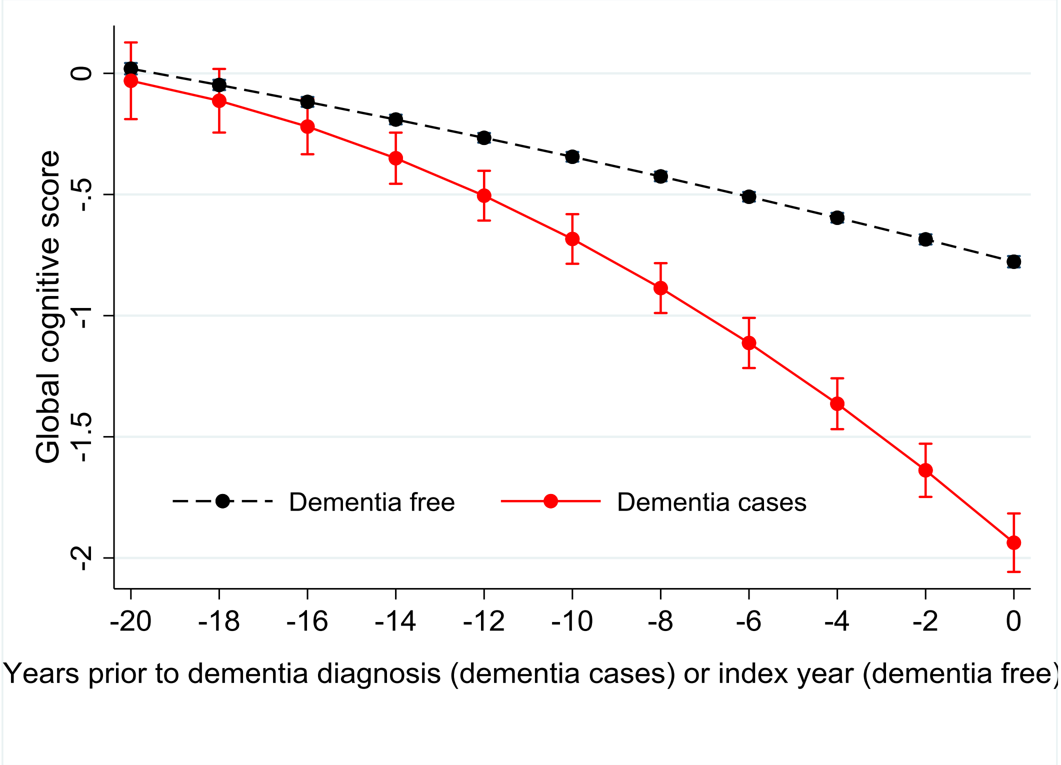

*To assess trajectories of cognitive function before dementia diagnosis, we used data from a cognitive test battery administered in 1997/99, 2002/04, 2007/09, 2012/13, and 2015/16. It consisted of tests of memory (20-word free recall test), reasoning (Alice Heim 4-I test composed of a series of 65 verbal and mathematical reasoning items of increasing difficulty), and verbal fluency (measures of phonemic (words starting with s) and semantic (animal names) fluency). A global cognitive score was created using all tests described above by first standardising the raw scores for each domain to z-scores (mean=0; standard deviation (SD)=1) using the distribution of the first wave of cognitive data. These z-scores were summed and restandardised to yield the global score.
To compare trajectories of global cognition in those with dementia compared to those free of dementia, a mixed model was used with a backward timescale such that Year=0 in the analysis was the year of dementia diagnosis for cases, year of death for those who died during the follow-up, and end of follow-up (March 31st 2017) for all others. The analysis was adjusted for age at time 0, sex, education, 5-year cohort of birth and dementia status and their interaction with time and time^2.

Figure S3. Role of midlife alcohol consumption in transitions to dementia and mortality: a multi-state model approach.

A. **All participants**

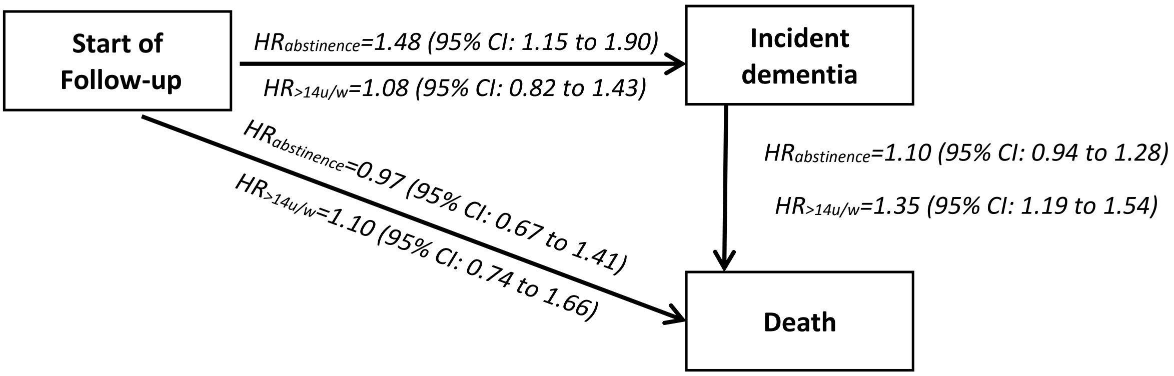

Analysis based on 9087 participants; N dementia=397; N death=1584 (among which 204 had dementia before death)
Model adjusted for age, sex, ethnicity, education, occupational position, and marital status.

B. **In participants with midlife alcohol consumption above 14 units/week**

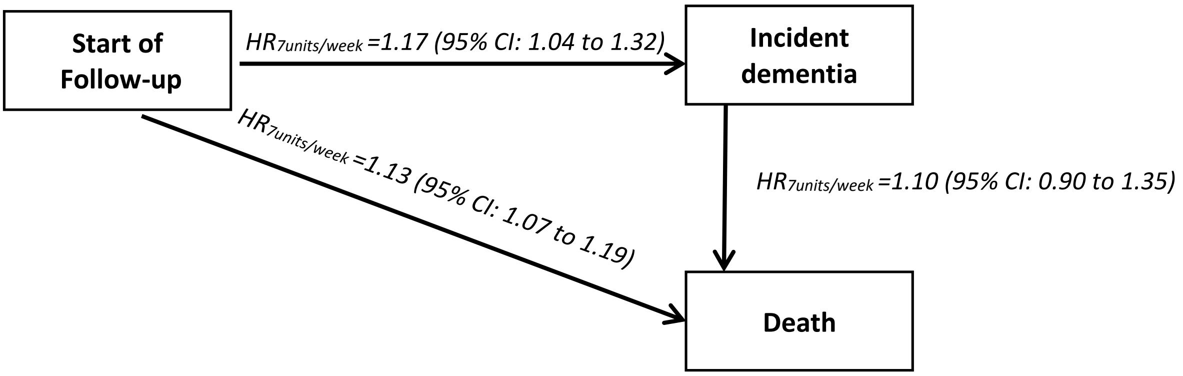

HRs correspond to an increment of 7 alcohol units per week.
Analysis based on 2232 participants; N dementia=70; N death=360 (among which 39 had dementia before death).
Analysis adjusted for age, sex, ethnicity, education, occupational position, and marital status.

Figure S4. Association between midlife alcohol consumption and incidence of dementia among drinkers* (A) and distribution of midlife alcohol consumption among drinkers (B).

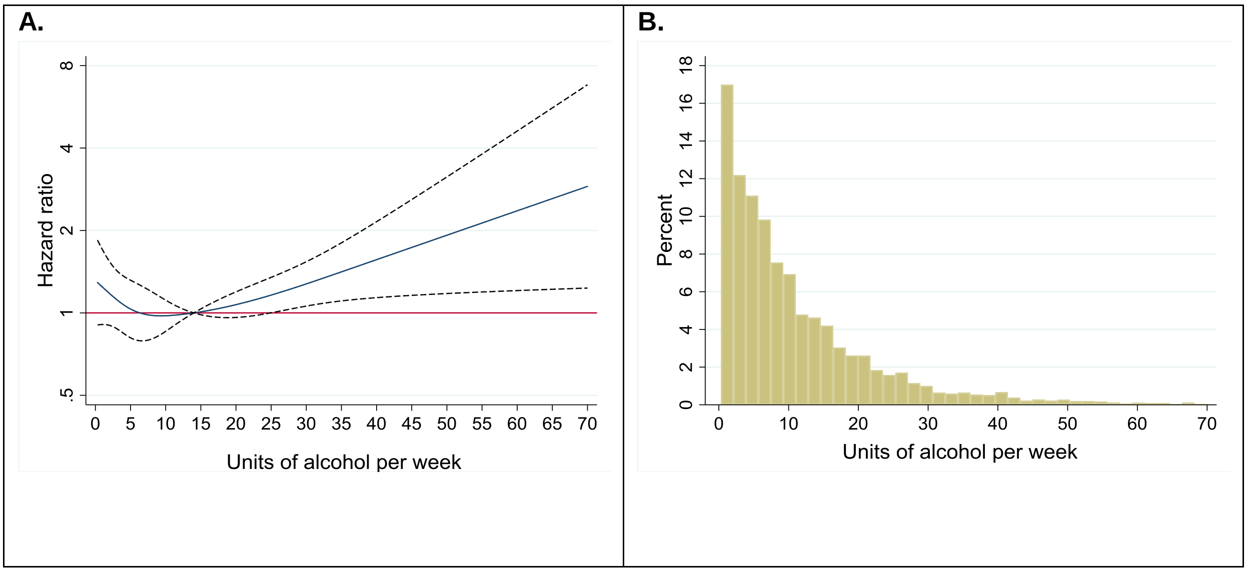

*Cox regression analysis adjusted for socio-demographic factors.

Figure S5. Association between midlife alcohol consumption and dementia by type of alcohol* (left panel) and corresponding distribution of types of alcohol consumed (right panel).

A. Association between wine and risk of dementia

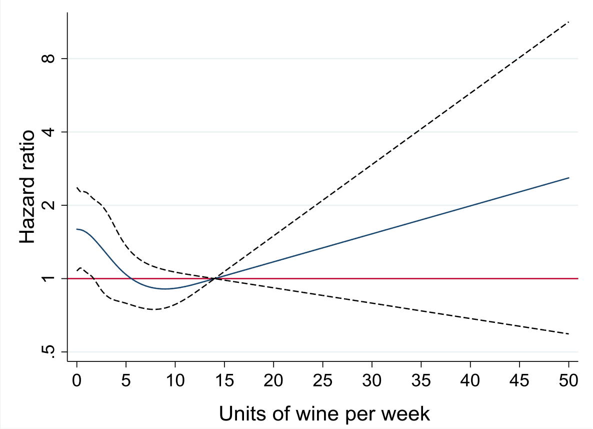

B. Distribution of wine consumption

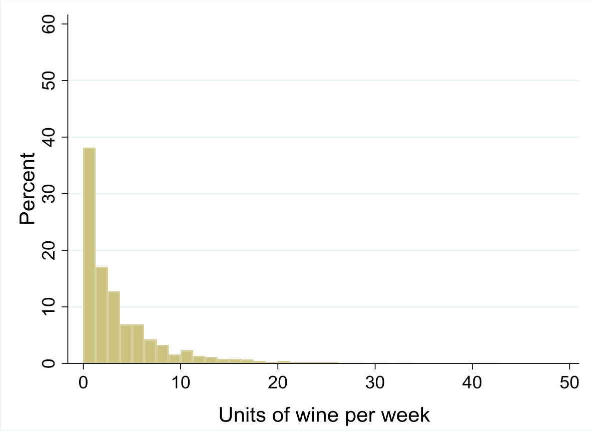

C. Association between beer and risk of dementia

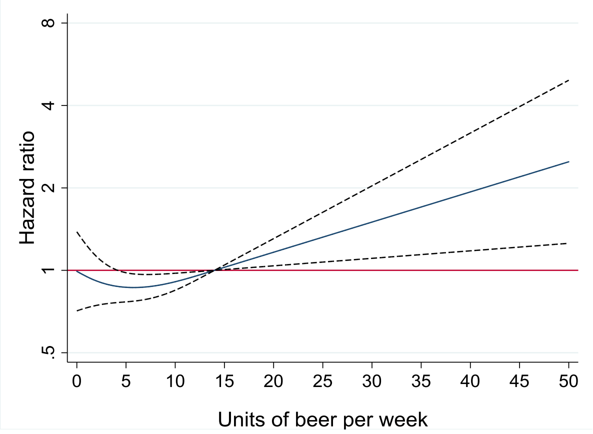

D. Distribution of beer consumption

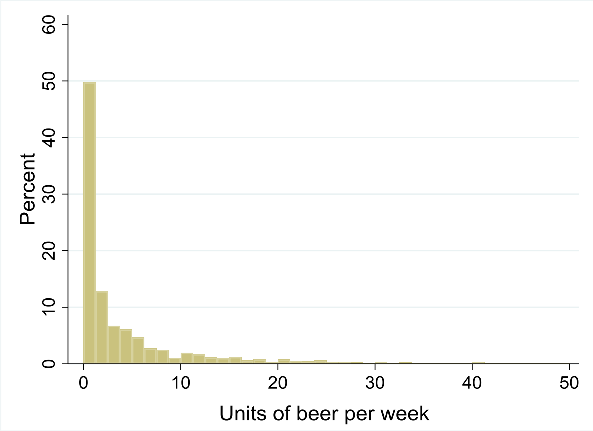

E. Association between spirit and risk of dementia

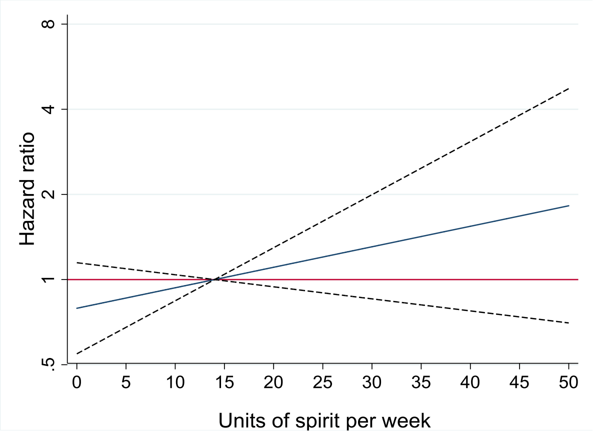

F. Distribution of spirit consumption

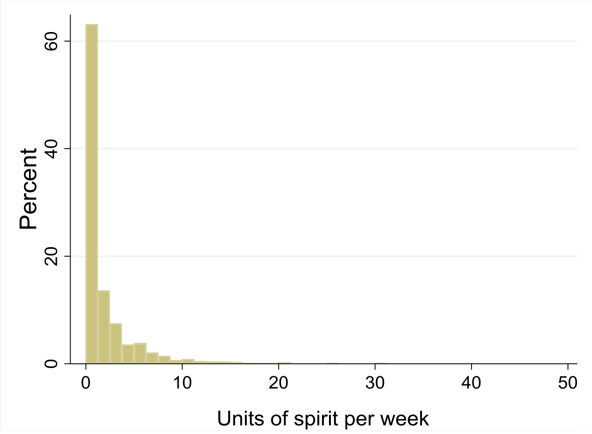

*Cox regression analysis adjusted for socio-demographic factors.

Table S1. Mortality and dementia incidence rates over time.

	Incidence of Mortality<br><br>Rate per 1000 person-years	Incidence of Dementia<br><br>Rate per 1000 person-years
**Analysis on midlife alcohol consumption**		
Included: N = 9087	7.2 (6.9 to 7.6)	1.9 (1.7 to 2.1)
Excluded: N = 1221	10.5 (9.4 to 11.9)	2.6 (2.0 to 3.3)
-N Dead = 77		
-N<2 alcohol measures = 1144		
**Analysis on CAGE**		
Included: N = 7969	6.7 (6.4 to 7.1)	1.8 (1.6 to 2.0)
Excluded: N = 2339	10.9 (10.0 to 11.8)	2.7 (2.3 to 3.2)
-N Dead = 77		
-N non response in 1991/93 = 2262		
**Analysis on alcohol-related chronic disease hospitalisation**		
Included: N = 10139	7.5 (7.2 to 7.9)	2.0 (1.8 to 2.1)
Excluded: N = 169	15.6 (10.9 to 22.4)	1.6 (0.5 to 4.9)
-N Dead = 77		
-N missing covariates = 92		
**Analysis on alcohol trajectories**		
Included: N = 8927	10.6 (10.0 to 11.2)	3.4 (3.1 to 3.8)
Excluded: N = 1381	13.8 (11.8 to 16.1)	4.6 (3.5 to 6.1)
-N prevalent dementia = 14		
-N dead = 491		
-N <2 alcohol measures = 876		

Table S2. Association between detailed categories of midlife alcohol consumption with incident dementia.

Midlife alcohol consumption	N cases/ N total	Hazard ratio (95% confidence interval)		
		Adjusted for socio-demographic variables†	Additionally adjusted for behavioural factors‡	Fully adjusted§
10y abstainers	22/269	1.60 (1.01 to 2.54)*	1.63 (1.03 to 2.58)*	1.73 (1.09 to 2.75)*
Former drinkers	12/172	1.37 (0.76 to 2.47)	1.38 (0.77 to 2.48)	1.21 (0.67 to 2.18)
Occasional drinkers	64/862	1.45 (1.09 to 1.94)*	1.46 (1.10 to 1.95)*	1.42 (1.06 to 1.90)*
1-14 units/week	229/5552	1 (ref)	1 (ref)	1 (ref)
>14 units/week	70/2232	1.08 (0.82 to 1.43)	1.05 (0.80 to 1.39)	1.02 (0.77 to 1.35)

*P<0.05
†Adjusted for age (time-scale), sex, ethnicity, education, occupational position, and marital status.
‡Additionally adjusted for physical activity, smoking status, and fruit and vegetable consumption.
§Additionally adjusted for systolic blood pressure, total cholesterol, diabetes, BMI, GHQ score, CVD, and CVD medication.

Table S3. Estimation of trajectories of alcohol consumption: model fit statistics (group based trajectory models).

Number of groups*	Trajectory shape†	Allocated Group membership	BIC‡	Average Posterior Probabilities§	AIC#
3	2<br>2<br>2	14.6%<br>64.2%<br>21.2%	-40336.25	0.93<br>0.97<br>0.94	-40293.67
4	2<br>2<br>2<br>2	14.6%<br>59.9%<br>11.2%<br>14.3%	-39195.94	0.93<br>0.96<br>0.92<br>0.97	-39139.16
**5**	**2**<br>**2**<br>**2**<br>**2**<br>**2**	**9.4%**<br>**5.6%**<br>**59.4%**<br>**11.2%**<br>**14.4%**	**-38720.77**	**0.96**<br>**0.90**<br>**0.95**<br>**0.92**<br>**0.97**	**-38649.80**
5	1<br>1<br>1<br>1<br>1	14.6%<br>11.3%<br>57.2%<br>4.7%<br>12.2%	-38931.74	0.92<br>0.89<br>0.96<br>0.87<br>0.95	-38863.52
5	1<br>1<br>1<br>1<br>2	14.6%<br>57.2%<br>4.7%<br>11.3%<br>12.2%	-38936.29	0.92<br>0.96<br>0.87<br>0.89<br>0.95	-38879.52
5	1<br>1<br>1<br>2<br>2	14.6%<br>57.2%<br>4.8%<br>11.2%<br>12.2%	-38755.81	0.92<br>0.96<br>0.88<br>0.92<br>0.95	-38695.49
5	1<br>1<br>2<br>2<br>2	14.6%<br>57.3%<br>11.2%<br>4.8%<br>12.2%	-38760.32	0.92<br>0.96<br>0.88<br>0.92<br>0.95	-38696.45
5	1<br>2<br>2<br>2<br>2	7.4%<br>9.9%<br>57.2%<br>11.2%<br>14.3%	-38821.62	0.92<br>0.86<br>0.94<br>0.92<br>0.97	-38754.20

* Number of trajectory groups estimated.
† Polynomial function of time (0 intercept only, 1 linear, 2 quadratic).
‡ Bayesian Information Criterion (BIC), a difference of 10 is strong evidence that the model with the lowest BIC (compared to null) has best fit.
§ Posterior probabilities of group membership for individuals assigned to each group, an average > 0.7 demonstrates good classification accuracy.
# Akaike Information Criterion (AIC).
Model selected based on fulfilment of criteria d and evidence of improved fit using lowest BIC/AIC score.

Table S4. ICD codes for identification of chronic diseases 100% attributable to alcohol consumption.

Cause	ICD-9	ICD-10
Alcoholic psychosis	291	F10.3-F10.9
Alcohol abuse	305.0, 303.0	F10.0, F10.1
Alcohol dependence syndrome	303.9	F10.2
Alcohol polyneuropathy	357.5	G62.1
Degeneration of nervous system due to alcohol	*	G31.2
Alcoholic myopathy	*	G72.1
Alcohol cardiomyopathy	425.5	I42.6
Alcoholic gastritis	535.3	K29.2
Alcoholic liver disease	571.0-571.3	K70-K70.4, K70.9
Fetal alcohol syndrome	655.4, 760.71	Q86.0
Fetus and newborn affected by maternal use of alcohol	*	P04.3, O35.4
Alcohol-induced chronic pancreatitis	*	K86.0

Table S5. Association between alcohol consumption at age 50, 60, and 70 and risk of dementia up to March 31st, 2017.

Variables	N cases/ N total	Hazard ratio (95% confidence interval)		
		Adjusted for socio-demographic variables†	Additionally adjusted for behavioural factors‡	Fully adjusted§
**ALCOHOL CONSUMPTION AT AGE 50 (N cases/N total=394/8878, Mean FU=23.1y (SD=6.2))**				
Abstainers	115/1649	1.43 (1.12 to 1.81)*	1.42 (1.12 to 1.82)*	1.38 (1.08 to 1.77)*
1-14 units/week	202/4906	1 (ref)	1 (ref)	1 (ref)
>14 units/week	77/2323	1.20 (0.92 to 1.58)	1.20 (0.91 to 1.58)	1.14 (0.86 to 1.50)
**Among those drinking above 14 units/week**				
Per 7 units/week increase	77/2323	1.10 (0.99 to 1.23)	1.09 (0.97 to 1.21)	1.09 (0.97 to 1.22)
**ALCOHOL CONSUMPTION AT AGE 60 (N cases/N total=369/8007, Mean FU=14.3y (SD=6.5))**				
Abstainers	114/1528	1.49 (1.16 to 1.90)*	1.51 (1.18 to 1.94)*	1.48 (1.15 to 1.89)*
1-14 units/week	176/4233	1 (ref)	1 (ref)	1 (ref)
>14 units/week	79/2246	1.19 (0.90 to 1.57)	1.19 (0.90 to 1.58)	1.19 (0.90 to 1.58)
**Among those drinking above 14 units/week**				
Per 7 units/week increase	79/2246	1.10 (0.97 to 1.23)	1.09 (0.97 to 1.23)	1.09 (0.96 to 1.23)
**ALCOHOL CONSUMPTION AT AGE 70 (N cases/N total=249/6542, Mean FU=5.8y (SD=4.7))**				
Abstainers	82/1539	1.53 (1.14 to 2.07)*	1.51 (1.12 to 2.04)*	1.41 (1.04 to 1.92)*
1-14 units/week	122/3483	1 (ref)	1 (ref)	1 (ref)
>14 units/week	45/1520	0.91 (0.64 to 1.29)	0.91 (0.64 to 1.29)	0.87 (0.61 to 1.23)
**Among those drinking above 14 units/week**				
Per 7 units/week increase	45/1520	1.06 (0.88 to 1.29)	1.06 (0.87 to 1.28)	1.07 (0.88 to 1.30)

Abbreviations: FU follow-up, SD: Standard deviation.
*P<0.05.
†Adjusted for age (time-scale), sex, ethnicity, education, occupational position, and marital status.
‡ Additionally adjusted for physical activity, smoking status, and fruit and vegetable consumption.
§ Additionally adjusted for systolic blood pressure, total cholesterol, diabetes, BMI, GHQ score, CVD, and CVD medication.

Table S6. Association between alcohol consumption and risk of mortality.

	N cases/ N total	Age at death	Adjusted for socio-demographic variables†
		Mean (SD)	HR (95%CI)
**Average alcohol consumption in midlife (1985/88, 1989/90 and 1991/93)**			
Abstinence	269/1303	70.7 (8.6)	1.15 (0.99 to 1.32)
1-14 units/week	885/5552	70.6 (8.7)	1 (ref)
>14 units/week	383/2232	68.1 (9.1)	1.31 (1.15 to 1.48)*
**Among those drinking above 14 units/week**			
Per 7 units/week increase	383/2232	68.1 (9.1)	1.12 (1.07 to 1.17)*

N cases/N total=1537/9087; mean FU=23.4y (SD=4.3).

† Adjusted for age (time-scale), sex, ethnicity, education, occupational position, and marital status.

Table S7. Sensitivity analysis to assess the impact of dementia misclassification on the association between alcohol consumption in midlife and dementia.*

	Correlation between classification indices	Abstainers vs 1-14 units/week<br><br>Odds ratio† (95% CI)	>14 units/week vs 1-14 units/week<br><br>Odds ratio† (95% CI)
**Observed data**	NA	1.49 (1.14 to 1.94)	1.01 (0.76 to 1.35)
**Simulated data: scenario 1**	0.8	2.05 (1.33 to 4.00)	0.75 (0.15 to 1.45)
**Simulated data: scenario 2**	0.6	2.07 (1.14 to 4.59)	0.75 (0.13 to 1.77)

***Simulated data were generated assuming sensitivity range between 60% and 90% and specificity range between 97% and 100% with a trapezoidal probability density function. This simulation makes the hypothesis of differential dementia misclassification (i.e. the hypothesis that probability of dementia misclassification depends on alcohol consumption). We first analysed the impact of potential differential misclassification on the risk in abstainers compared to those drinking 1-14 alcohol units/week. We simulated two scenarios: first where the correlation between the sensitivity distribution in those following recommendations and the sensitivity distribution in all others was 0.8 and then 0.6 (and similarly for the correlations of the specificity distributions); note that a correlation of 1 corresponds to non-differential misclassification. We then repeated these analyses for alcohol consumption >14 units/week compared to 1-14 units/week.

† Adjusted for sociodemographic variables

Abbreviation: NA, not applicable.
